# Second-line treatment for advanced NSCLC without actionable mutations: is immunotherapy the ‘panacea’ for all patients?

**DOI:** 10.1186/s12916-018-1011-0

**Published:** 2018-02-16

**Authors:** Alessandro Morabito

**Affiliations:** The Medical Oncology Unit, Thoracic-Pulmonary Department, Istituto Nazionale Tumori, “Fondazione G. Pascale” – IRCCS, Via Mariano Semola, 80131 Naples, Italy

**Keywords:** Nivolumab, Pembrolizumab, Atezolizumab, Afatinib, Nintedanib, Ramucirumab, Docetaxel, Pemetrexed, Erlotinib, NSCLC, Second-line therapy

## Abstract

The therapeutic approach for the second-line treatment of patients with advanced non-small cell lung cancer (NSCLC) without actionable mutations has been revolutionized by the recent approval of new effective drugs with various mechanisms of action, including nintedanib, ramucirumab, nivolumab, pembrolizumab, atezolizumab, and afatinib. The recent network meta-analysis of Créquit et al. (*BMC Medicine*, 15:193, 2017) compared the effectiveness and tolerability of the second-line treatments for advanced NSCLC with wild-type or unknown status for EGFR. The authors found that immunotherapy might be more efficacious than the currently recommended treatments. However, their meta-analysis does not take into account the role of predictive biomarkers – this is indeed a crucial point in the decision-making process considering that only a fraction of advanced NSCLC patients might derive a long-term benefit from second-line immunotherapy. The identification of molecular biomarkers that can predict a response to immune checkpoints, angiogenesis, and EGFR inhibitors remains an important goal of clinical research in order to maximize the benefit of these agents and to aid clinicians in the decision-making process.

Please see related article: https://bmcmedicine.biomedcentral.com/articles/10.1186/s12916-017-0954-x

## Background

The recommended therapeutic options for the second-line treatment of patients with advanced non-small cell lung cancer (NSCLC) without actionable mutations has, until recently, mainly included docetaxel, pemetrexed (only for non-squamous histology), and erlotinib [1, 2]. This therapeutic approach has now been revolutionized by the approval of new effective drugs with various mechanisms of action, including angiogenesis, immune checkpoint, and EGFR inhibitors (Table 1) [3–9]. In patients with non-squamous histology, nintedanib or ramucirumab plus docetaxel, nivolumab, atezolizumab, and pembrolizumab (in patients with programmed death ligand-1 (PD-L1) > 1%) prolonged overall survival compared to docetaxel single agent [3, 4, 6–8]. In patients with squamous histology, ramucirumab plus docetaxel, nivolumab, atezolizumab, pembrolizumab (in patients with PD-L1 > 1%), or afatinib were more efficacious than docetaxel or erlotinib [4, 5, 7–9]. Therefore, with the increasing number of available therapeutic options and patients approaching a second-line therapy, the therapeutic scenario has become more complex and the choice of the best second-line treatment is proving a significant challenge for oncologists.

**Table 1:** New approved drugs for the second-line treatment of patients with advanced NSCLC

Reference	Patients	Histotype	Regimen	Response	Progression-free survival, months	Median overall survival, months	*P*
Reck et al. [3]	658	Adenocarcinoma	Docetaxel + nintedanib vs. docetaxel + placebo	4.7% vs. 3.6%	4.0 vs. 2.8	12.6 vs. 10.3	0.0359
Garon et al. [4]	1253	All histologies	Docetaxel + ramucirumab vs. docetaxel + placebo	23% vs. 14%	4.5 vs. 3.0	10.5 vs. 9.1	0.023
Brahmer et al [5]	272	Squamous	Nivolumab vs. docetaxel	20% vs. 9%	3.5 vs. 2.8	9.2 vs. 6.0	<0.001
Borghaei et al. [6]	582	Adenocarcinoma	Nivolumab vs. docetaxel	19% vs. 12%	2.3 vs. 4.2	12.2 vs. 9.4	0.002
Herbst et al. [7]	1034	All histologies	Pembrolizumab 2 mg/kg vs. pembrolizumab 10 mg/kg vs. docetaxel	18% vs. 18% vs. 9%	3.9 vs. 4.0 vs. 4.0	10.4 vs. 12.7 vs. 8.5	0.0008 < 0.0001
Rittmeyer et al. [8]	287	All histologies	Atezolizumab vs. docetaxel	14% vs. 13%	2.8 vs. 4.0	13.8 vs. 9.6	0.0003
Soria et al. [9]	795	Squamous	Afatinib vs. erlotinib	6% vs. 3%	2.6 vs. 1.9	7.9 vs. 6.8	0.0077

### Network meta-analysis of second-line treatments

In the network meta-analysis of Créquit et al. [10], the authors compared the effectiveness and tolerability of the second-line treatments for advanced NSCLC with wild-type or unknown status for EGFR. Nivolumab, pembrolizumab, atezolizumab, and pemetrexed plus erlotinib were shown to be significantly more effective in terms of overall survival than docetaxel, pemetrexed, erlotinib, or gefitinib, and together with erlotinib plus cabozantinib represented the five most effective treatments in terms of overall survival. Indeed, the ‘old’ four recommended treatments were ranked in the 30th position, with no difference in effectiveness between them being observed. The authors’ main conclusion was that immunotherapy is more efficacious than the current recommended treatments in the second-line treatment of patients with NSCLC without actionable mutations.

Nevertheless, a major limitation of this network meta-analysis was the inclusion of only a small number of trials designed in a population of patients selected for biomarkers. Therefore, the predictive role of biomarkers, which is indeed a crucial point in the decision-making process, was not considered. Currently, only a fraction of advanced NSCLC patients might derive a long-term benefit from second-line immunotherapy.

### Predictive biomarkers

In patients with non-squamous histology, the CheckMate-057 study demonstrated a longer overall survival with nivolumab compared with single agent docetaxel (12.2 vs. 9.4 months, HR 0.73, *P* = 0.002), but patients with poorer prognostic factors and/or more aggressive disease combined with lower or no PD-L1 expression appeared to be at higher risk of death within the first 3 months on nivolumab versus docetaxel [11]. Exploratory analyses suggested that higher levels of PD-L1 were associated with a greater magnitude of overall survival benefit with nivolumab [12]. The role of PD-L1 as a predictive biomarker has also been demonstrated for pembrolizumab and atezolizumab, as confirmed by recent meta-analyses [7, 8, 13, 14]. However, there are a number of PD-L1 testing limitations that can confound its use as a predictive biomarker, including the heterogeneity and dynamics of PD-L1 expression, the varying performance of the various immunohistochemistry-based assays with different cutoffs, the absence of consensus regarding the relevance of geographic patterns of expression of PD-L1 or its expression on tumor or inflammatory cells within the tumor microenvironment and, finally, the availability of an adequate sample [15].

On the other hand, the LUME-Lung-1 study [3] showed that nintedanib (a triple angiokinase inhibitor) plus docetaxel significantly improved overall survival in pretreated patients with adenocarcinoma histology (12.6 vs. 10.3 months, HR 0.83, *P* = 0.0359), with a greater survival advantage in patients with early progression of disease or refractory to first-line therapy, or with a greater tumor burden, as well as a non-negligible number of ‘long-surviving’ patients (over 32 months).

**Fig. 1:**
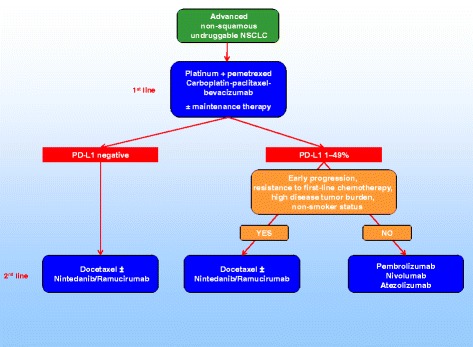
Therapeutic scenario for patients with advanced non-squamous ‘undruggable’ NSCLC (EGFR wild type, ALK and ROS1 non-rearranged, PD-L1 < 50%)

Therefore, both molecular and clinical criteria should be considered in the decision-making tree of non-squamous NSCLC patients, including PD-L1 expression and clinical factors associated with higher probability of response to nintedanib plus docetaxel (early progression or resistance to first-line therapy, high disease burden) or lower probability of response to nivolumab (progression disease as best response to prior treatment, early progression, five or more sites with lesions, bone or hepatic metastases, non-smoker status) (Fig. 1) [16, 17].

**Fig. 2:**
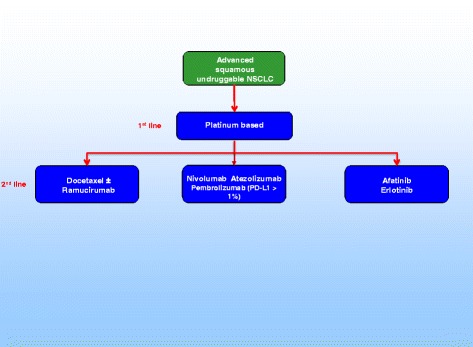
Therapeutic scenario for patients with advanced squamous ‘undruggable’ NSCLC (PD-L1 < 50%)

In patients with squamous histology, the CheckMate-017 study demonstrated the superiority of nivolumab over docetaxel regardless of PD-L1 expression level [5]. However, in this setting, there are additional options of treatment represented by afatinib or ramucirumab plus docetaxel (Fig. 2). In the Lux-Lung-8 study [9, 18], afatinib, an irreversible inhibitor of multiple members of the EGFR family, was superior to erlotinib, with 5% of patients being long-term responders (median overall survival of nearly 2 years). Exploratory analyses are ongoing to better define the molecular characteristics of patients associated to prolonged survival and, to date, a Veristrat-Good serum protein test and the presence of ErbB family mutations have been highlighted as potential biomarkers of long-term response to afatinib [19, 20]. In the REVEL study [4], ramucirumab, a totally humanized IgG1 monoclonal antibody that specifically targets the extracellular domain of VEGFR2, plus docetaxel was superior to docetaxel single agent, although it was associated with a worst toxicity profile. This is the first evidence supporting the use of an angiogenesis inhibitor also in patients with squamous histology. Unfortunately, to date, there are no validated biomarkers that could predict response to ramucirumab as well as nintedanib.

Therefore, the identification of proper predictive biomarkers for immunotherapy, angiogenesis, or EGFR inhibitors remains a crucial point in the era of precision medicine, and would likely contribute to the optimization of patient or treatment selection; this should be pursued in future studies.

### Future implications for clinical practice

PD-L1, notwithstanding all its limitations, is to date the only molecular factor able to guide the choice of a second-line therapy for patients with advanced non-squamous non-oncogene-addicted NSCLC. A number of additional factors are under investigation, including the tumor mutational burden, tumor-infiltrating lymphocytes, indoleamine 2,3-dioxygenase, DNA mismatch repair deficiency, and the expression of inflammatory genes. Moreover, by examining histological sections of tumor biopsies collected from patients prior to receiving immunotherapy, three basic immune profiles that correlate with response to anti-PD-L1/PD-1 therapy have been described, namely an immune-inflamed phenotype generally correlated with higher response rates to anti-PD-L1/PD-1 therapy, an immune-excluded phenotype associated with uncommon clinical responses, and an immune-desert phenotype rarely responsive to anti-PD-L1/PD-1 therapy [21]. Finally, emerging data also suggests that a subset of patients even appears to experience a tumor flare under checkpoint inhibitors, which has been recognized as a novel aggressive pattern of disease termed hyper-progression [22]. A hyper-progression can be observed in roughly 10% of immunotherapy-treated patients and specific genomic alterations, e.g., the presence of MDM2 family amplification or EGFR aberrations, seem to be associated with this clinical feature [23].

## Conclusions

In conclusion, the network meta-analysis of Créquit et al. [10] showed that immunotherapy might be more efficacious than the current recommended treatments in the second-line therapy of NSCLC; nevertheless, immunotherapy cannot be considered the ‘panacea’ for all NSCLC patients.

Currently, both clinical and molecular criteria (to date by detection of PD-L1) should be considered in the definition of the best therapeutic approach of patients with pre-treated NSCLC without actionable mutations. In the future, genomic and immune profiles will help to identify patients eligible for immunotherapy.
